# Multilayered Networks of SalmoNet2 Enable Strain Comparisons of the Salmonella Genus on a Molecular Level

**DOI:** 10.1128/msystems.01493-21

**Published:** 2022-08-01

**Authors:** Marton Olbei, Balazs Bohar, David Fazekas, Matthew Madgwick, Padhmanand Sudhakar, Isabelle Hautefort, Aline Métris, Jozsef Baranyi, Robert A. Kingsley, Tamas Korcsmaros

**Affiliations:** a Earlham Institute, Norwich, United Kingdom; b Quadram Institute Biosciences, Norwich, United Kingdom; c Eotvos Lorand University, Budapest, Hungary; d KU Leuven, Leuven, Belgium; e Institute of Nutrition, University of Debrecen, Debrecen, Hungary; f School of Biological Sciences, University of East Anglia, Norwich, United Kingdom; University of California, San Francisco

**Keywords:** host adaptation, *Salmonella*, global regulatory networks, network resource, protein-protein interactions

## Abstract

Serovars of the genus Salmonella primarily evolved as gastrointestinal pathogens in a wide range of hosts. Some serotypes later evolved further, adopting a more invasive lifestyle in a narrower host range associated with systemic infections. A system-level knowledge of these pathogens could identify the complex adaptations associated with the evolution of serovars with distinct pathogenicity, host range, and risk to human health. This promises to aid the design of interventions and serve as a knowledge base in the Salmonella research community. Here, we present SalmoNet2, a major update to SalmoNet1, the first multilayered interaction resource for Salmonella strains, containing protein-protein, transcriptional regulatory, and enzyme-enzyme interactions. The new version extends the number of Salmonella networks from 11 to 20. We now include a strain from the second species in the Salmonella genus, a strain from the Salmonella enterica subspecies *arizonae* and additional strains of importance from the subspecies *enterica*, including S. Typhimurium strain D23580, an epidemic multidrug-resistant strain associated with invasive nontyphoidal salmonellosis (iNTS). The database now uses strain specific metabolic models instead of a generalized model to highlight differences between strains. The update has increased the coverage of high-quality protein-protein interactions, and enhanced interoperability with other computational resources by adopting standardized formats. The resource website has been updated with tutorials to help researchers analyze their Salmonella data using molecular interaction networks from SalmoNet2. SalmoNet2 is accessible at http://salmonet.org/.

**IMPORTANCE** Multilayered network databases collate interaction information from multiple sources, and are powerful both as a knowledge base and subject of analysis. Here, we present SalmoNet2, an integrated network resource containing protein-protein, transcriptional regulatory, and metabolic interactions for 20 Salmonella strains. Key improvements to the update include expanding the number of strains, strain-specific metabolic networks, an increase in high-quality protein-protein interactions, community standard computational formats to help interoperability, and online tutorials to help users analyze their data using SalmoNet2.

## INTRODUCTION

Serovars of the genus Salmonella are enteric pathogens, capable of causing a self-limiting gastrointestinal inflammatory disease in a variety of animals. The host species ranges from cold-blooded vertebrates to mammals, depending on the Salmonella species, subspecies, and strain. Salmonella infection is one of the most common foodborne or waterborne illnesses resulting in approximately 94 million cases of illness and 155,000 deaths each year (1–3).

Of six subspecies of Salmonella enterica, a small number of subspecies I serovars have adapted to cause an invasive infection in a restricted host range, instead of a self-limiting gastrointestinal inflammation typical of most Salmonella serovars. These extraintestinal Salmonella strains, including the human adapted typhoidal strains, emerged on multiple occasions independently. The hallmarks of adaptation are genomic and phenotypic changes, including loss of function mutations in genes related to adaptation to specific niches in their host commonly affecting anaerobic metabolism, virulence genes, chemotaxis, or motility (4).

Salmonella Typhi is an ancient pathogen and the most common extraintestinal Salmonella serovar to infect humans. Over the past decades, invasive nontyphoidal Salmonella (iNTS) emerged as one of the most commonly isolated pathogens from the blood of patients, particularly in sub-Saharan Africa (5). The invasive nontyphoidal Salmonella (iNTS) strains, in common with S. Typhi and *S.* Paratyphi, cause a systemic infection, but unlike S. Typhi, iNTS commonly affects immunocompromised individuals and young children, leading to bacteremia and meningitis. iNTS is most often caused by specific genotypes of S. Typhimurium and S. Enteritidis that are distinct from genotypes of these serovars commonly associated with gastrointestinal infections outside sub-Saharan Africa (6–8).

The Salmonella genus contains pathogens with diverse host range and pathogenicity, and dissecting the specific differences between gastrointestinal and extraintestinal strains have been pursued by a multitude of means (4, 9, 10). Previously, we developed SalmoNet1, the first public multilayered network resource for Salmonella research. SalmoNet1 is a network resource containing integrated information on the protein-protein, regulatory, and metabolic interactions of 10 Salmonella serovars (11). With its multilayered networks, SalmoNet1 has served as a knowledge base for the community and aided in understanding Salmonella pathogenesis and evolution by mapping the differences in molecular interactions between Salmonella pathovars on multiple biological levels. The systems-level information of SalmoNet1 allows researchers to enhance the information content of their own studies, by adding interaction context to the changes observed on a genomic or transcriptome level.

Here, we present SalmoNet2, that extends the coverage of strains from 11 to 20, including an important iNTS strain, and strains outside subspecies *enterica*, from subspecies *arizonae* and Salmonella bongori. To aid interoperability in computational biology, the database adopted the proteomics standard initiative-molecular interactions (PSI-MI TAB) format, and is now accessible through the NDEx (The Network Data Exchange) network repository (12, 13). In addition, we show how rewiring of the network information can be utilized by the research community to understand aspects of Salmonella evolution. As part of our update, we created step-by-step workflows with tutorials to help the Salmonella community use the resource, accessible on our website (http://salmonet.org/).

## RESULTS

### SalmoNet2 extends the list of included strains.

SalmoNet2 adds nine new multilayered networks of Salmonella strains to the database compared with the first version. Among the new additions is a strain of Salmonella bongori, another species in the Salmonella genus. We also inserted a strain from a different subspecies of S. enterica, subsp. *arizonae*. Additionally, we included two regularly used laboratory strains and four extraintestinal strains, including S. Typhimurium strain D23580, a well-characterized pathogen linked with invasive nontyphoidal Salmonella (iNTS) disease. The extended strain coverage captures a larger variety of the Salmonella genus, and provides interaction networks sampling a larger diversity of the Salmonella genus (Table S1).

10.1128/msystems.01493-21.1 TABLE S1 List of strains in SalmoNet2, and the overlap of the orthologous proteins with that of Escherichia coli, used as a measure of recall. Download Table S1, PDF file, 0.07 MB. Copyright © 2022 Olbei et al. 2022 Olbei et al. https://creativecommons.org/licenses/by/4.0/ This content is distributed under the terms of the Creative Commons Attribution 4.0 International license.

**FIG 1:**
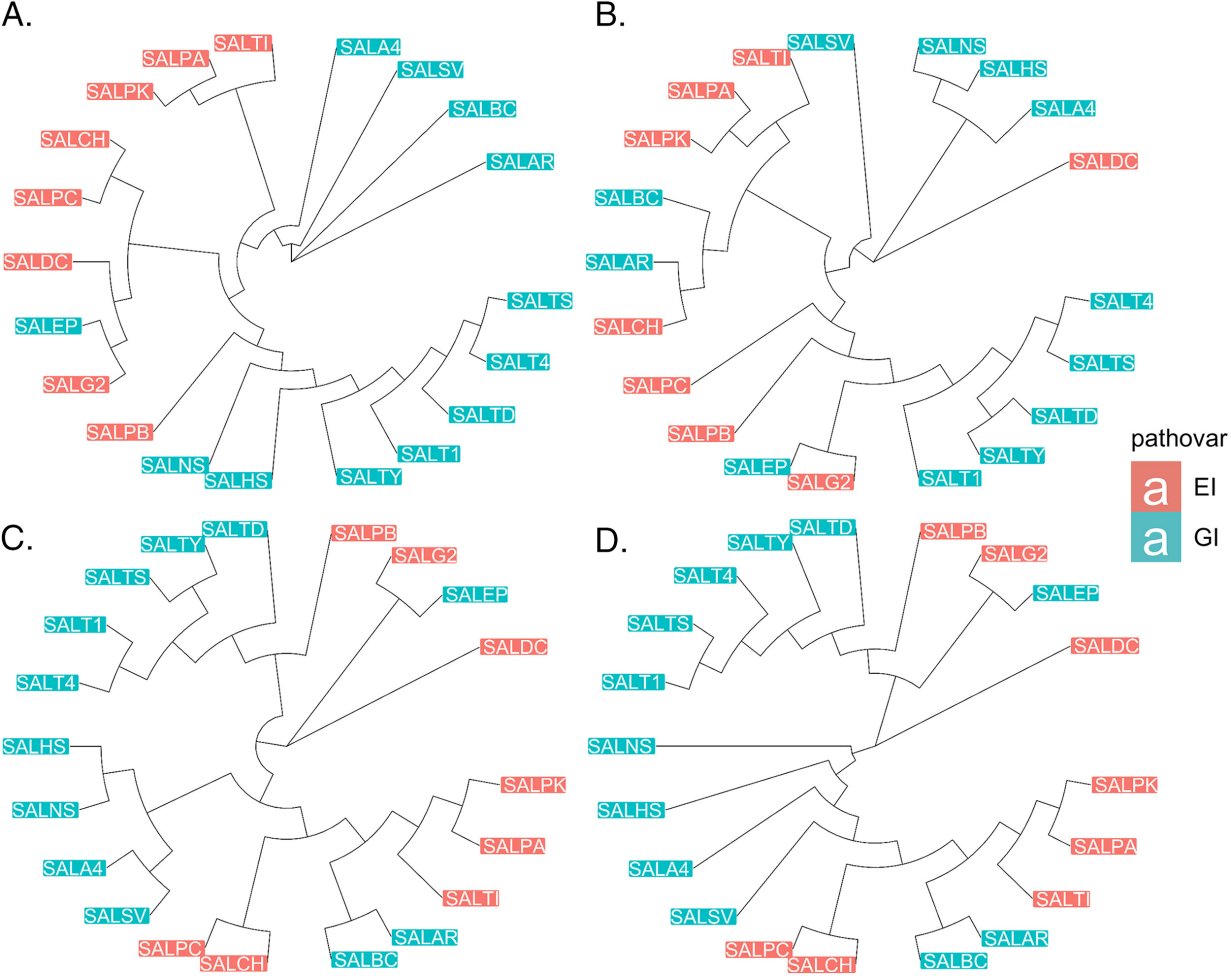
Core genome SNP based phylogenetic tree, and hierarchical classification of network layers. Extraintestinal (EI) serovars labeled with red, gastrointestinal (GI) serovars with blue labels. (A) Neighbor-joining tree from core genome single-nucleotide polymorphisms (SNPs) of the strains. (B to D) Hierarchical classification trees based on matrix representation of protein-protein, regulatory, and metabolic networks. The five letter labels encode the names of the different strains (for details of the encoding please refer to Table S1).

We constructed a neighbor-joining tree from variation in the core genome nucleotide sequence to define the phylogenetic relationship of the strains included in the database. The resulting phylogeny was compared with hierarchical classification trees based on matrix representation of the protein-protein, regulatory, and metabolic networks of the included Salmonella strains (Fig. 1).

The topology was in accordance with previously published phylogenies (14). While some extraintestinal serovars clustered on closely related terminal branches, these were present on multiple major lineages suggesting emergence from ancestors that caused a gastrointestinal infection. This was consistent with previous reports that extraintestinal serovars of Salmonella are not monophyletic (15, 16). A PERMANOVA analysis of the distance matrices highlighted that out of the four different layers of information, the metabolic layer predicts best the pathovar status (R2: 0.32658, *P*-value: 0.0011).

### SalmoNet2 increases the information content of the individual network layers.

We included a number of methodological improvements to the workflow of the SalmoNet1 database leading to an increased number of high-quality interactions for individual network layers. In SalmoNet2, we used the OMA (“Orthologous Matrix”) standalone software to construct the orthologous relationships between the available Salmonella strains from the OMA browser database (17). OMA is a large-scale orthology database and toolkit, containing much of the information needed for SalmoNet2 in one place (18). The reason for this change was the ease of use and extra information provided by the OMA browser database. This makes it easier to generate minor and major releases for the future.

Given that Salmonella is a nonmodel organism, due to the lack of specific resources, some of the interactions that were included rely on interolog predictions from model organisms, such as the commensal bacteria Escherichia coli (19). To increase the coverage of the protein-protein interactions (PPIs) in SalmoNet2 without compromising quality, we have used the IntAct PSI-MIscore when extrapolating orthologous E. coli interaction information from the IntAct database (20). The IntAct PSI-MIscore assigns scores to interactions based on interaction detection method, interaction type, and the number of publications the interaction has appeared in, normalized between 0 and 1. Thus, instead of relying on one experimental method as in SalmoNet1, using the IntAct PSI-MIscore as a quality filter we could extend the number of available high-quality protein-protein interactions from E. coli (Fig. S1).

10.1128/msystems.01493-21.2 FIG S1 Distribution of the PSI-MIscores in the E. coli IntAct dataset. 0.50 was used as a cutoff for inclusion. Download FIG S1, PDF file, 0.2 MB. Copyright © 2022 Olbei et al. 2022 Olbei et al. https://creativecommons.org/licenses/by/4.0/ This content is distributed under the terms of the Creative Commons Attribution 4.0 International license.

For SalmoNet1, we built enzyme-enzyme interaction networks using a well-established genome scale metabolic model described previously (21). In contrast, SalmoNet2 utilizes strain-specific genome-scale metabolic models developed for each strain separately by Seif et al. (22). As a result, the metabolic layer now includes more enzyme-enzyme relationships, where two proteins are connected if a metabolite produced by one is a substrate for the other (21–23), leading to a more complete description of the metabolic capabilities of the strains.

For SalmoNet2, the information content of position-specific scoring matrices (PSSMs) that are required to carry out genome-wide regulatory scans, were enhanced with novel binding sites published since SalmoNet1, and from new data uploaded to the CollecTF repository (24).

**FIG 2:**
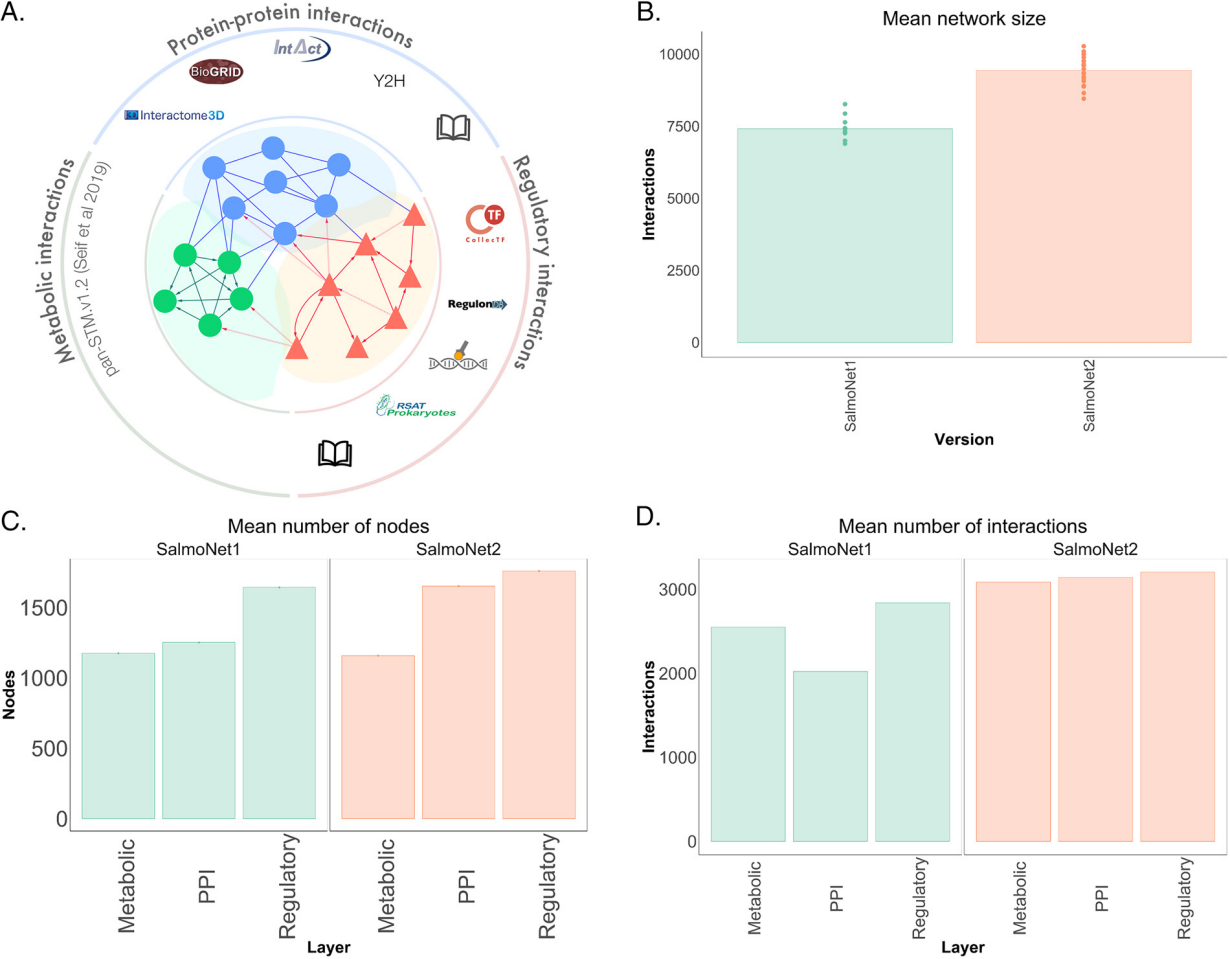
Comparison of SalmoNet1 and 2. (A) Main data sources and interactions in SalmoNet2. (B) Comparison of network size in SalmoNet1 and SalmoNet2. (C) Comparison of layer size in terms of participating nodes. (D) Comparison of layer size in terms of interactions between SalmoNet1 and SalmoNet2. The five letter codes encoding the different strains can be found in Table S1.

The total number of interactions has increased from 81,514 to 190,461, primarily due to the expansion of the PPI layer, and the increase in the number of strains. The composition of the consensus network, comprised of shared interactions among all strains included in the database, slightly changed from SalmoNet1, indicating the shifts caused by the updated data sources and expanded strain repertoire. In total, 24.4% of regulatory interactions (up from 16%), 68.1% of PPIs (down from 72%), and 51.8% of metabolic interactions (down from 69%) were shared among all strains, forming the core network of Salmonella interactions. Fig. 2 shows the changes in the size of the networks and individual layers compared to the first version.

### Novel formats improve interoperability.

In addition to the formats used previously (comma-separated values [.csv] used with spreadsheet software such as Microsoft Excel, and .cys the network file format used by Cytoscape), we extended the output format data to include the standardized format PSI-MI TAB, commonly used in computational biology. Using standardized formats improves the interoperability with other network information repositories, and provides space to maximize each interaction with as much data as possible, in a controlled and transparent manner (12). PSI-MI TAB helps computational biologists access network information in SalmoNet2, contains a strictly regulated vocabulary for interaction data, and is a prerequisite for the inclusion in the PSICQUIC ecosystem, which would further increase the potential reach of SalmoNet2 (12).

To enable the networks to be directly accessible from the widely used Cytoscape network analysis program, we have also deposited them to the NDEx network repository (25). This further strengthens the accessibility of SalmoNet2, and improves the user experience, as no additional files have to be downloaded.

### Website enhancements for a user-friendly experience.

The SalmoNet2 website has been enhanced compared with SalmoNet1. SalmoNet2 now carries new locus tag identifiers for all Salmonella strains to enable users to map their experimental data to the SalmoNet2 interaction networks. We have also mapped KEGG identifiers to all metabolic enzymes with the same goal in mind. We also included changes to help users traverse the website: the interacting proteins can now be accessed by simply clicking on the nodes in the graphs shown on the website.

SalmoNet2 now directly links to the respective OMA pages and sequence data instead of Uniprot (18), but where possible, Uniprot data are still accessible through OMA (26).

Since the introduction of SalmoNet1, we identified a potential bottleneck for our users. The interaction network format, while useful for scientists with a microbiology background, proved difficult to use. We expected that in time there could be fewer research groups using the resource due to this. To resolve this problem, we have created a new tab on the website containing tutorials using the SalmoNet2 database. These tutorials serve as an introduction to network analysis, and contain step-by-step workflows to help carry out analyses such as the one shown in this article.

While researchers currently cannot incorporate genomes of interest using the website for now, we did create a separate tutorial repository that includes an example on how researchers can generate their own interaction networks. We include helper scripts to deal with the output of OMA, and to add various interaction layers based on orthology, either from E. coli, or better-described Salmonella strains, such as S. Typhimurium LT2. We plan to add additional tutorials, workflows, and examples to the website in the future, to further increase the usability and longevity of the platform.

### Case study: analyzing network rewiring to identify functional differences in S. enterica.

Network rewiring entails many approaches aimed at quantifying changes between interaction networks, and has been used to identify differences between interaction networks (27, 28). A rewired node and its rewiring score sums up the quantitative (i.e., how many interactors does the node have) and qualitative (i.e., other interacting nodes) differences between the same node (i.e., same transcription factor, same protein, etc.) across different networks. Using this, we can analyze the differences in interaction networks node-by-node, and highlight which neighborhoods are changing the most between the compared strains.

To explore the utility of the SalmoNet2 multilayered network resource, we calculated the rewiring scores for all nodes between the interactomes of four host adapted typhoidal Salmonella strains and four gastrointestinal Salmonella strains. We also compared the most rewired nodes and their first neighbors from subgraphs of the typhoidal and gastrointestinal Salmonella strains to find the specific differences in interactions that led to the high rewiring score, using degree corrected rewiring values from the DyNet Cytoscape package (29).

In general, the most rewired nodes were global regulators, such as Crp, Fis, and Fur. The significantly enriched functions of their first neighbors are similar between the compared strains, with a few key differences. For example, the ferric uptake regulator Fur senses metal concentration and redox state of cells, and regulates many operons and genes involved in these processes (30). Interestingly, Fur and its first neighbors are enriched in the Gene Ontology function “iron ion homeostasis” in all four gastrointestinal strains tested, while this was not observed for the typhoidal strains. The absent function is explained by the absence of Fur interactions with the genes *fhuA and fhuE* that encode TonB-dependent receptors for the uptake of siderophores in typhoidal strains (31). The loss of these interactions is caused by the disruption of coding sequences in these genes in typhoidal serovars, as highlighted previously in the literature (15, 32). Similarly, the Fur subgraph is enriched in the term “cell adhesion” in all gastrointestinal strains, whereas this function is missing in typhoidal strains, with the exception of *S.* Paratyphi C. Similar to the rewiring of *fhuA* and *fhuE*, the difference in functional enrichment is due to the pseudogenization and subsequent missing interactions with the genes *stiH* and *stiA* in the rest of the typhoidal Salmonella strains. StiH and StiA are responsible for the production of fimbriae, highlighted previously in the literature (15).

From the top 50 most rewired nodes, an average of 33 nodes had at least one pseudogene first neighbor in the typhoidal serovars, and an average of 4% of the first neighbors of the top 50 most rewired nodes were pseudogenes. In the gastrointestinal strains, an average of seven nodes had pseudogene first neighbors, and only 1% of their first neighbors were pseudogenes.

While a large part of the rewiring was due to gene loss in typhoidal and extraintestinal serovars, we found examples where the cause of rewiring was due to the exclusivity of genes to the extraintestinal group. Two proteins, YreP and YjcS, are present in all typhoidal and extraintestinal strains of Salmonella included in SalmoNet2. However, they are missing from all gastrointestinal strains but one; protein YjcS has an orthologue in S. Enteritidis, but YjcS is otherwise missing from the gastrointestinal group. These two genes share an upstream regulatory region, and are predicted to interact with the regulators HilC, RtsA, and Fur. They were first described together in E. coli, in two analyzed strains: E. coli SMS-3-5 and E. coli (NMEC) O7:K1 strain CE10. *YreP* encodes a putative diguanylate cyclase, based on the presence of a GGDEF domain (33, 34). Diguanylate-cyclases facilitate the production of c-di-GMP, a ubiquitous secondary messenger metabolite in prokaryotes (33, 34). The second gene, *yjcS*, is an alkyl-sulfatase. This enzyme was first described in Pseudomonas spp., where a strain carrying this enzyme was able to grow on the surfactant sodium dodecyl sulfate (SDS), and the gene has been characterized in E. coli as well (35, 36).

After noting their presence in the extraintestinal strains included in SalmoNet2, we expanded the search into a more expansive data source. We ran a BLAST search against the pubMLST database to see if this split was representative of the serovars as a whole, and not just the specific strains in SalmoNet2 (37).

**FIG 3:**
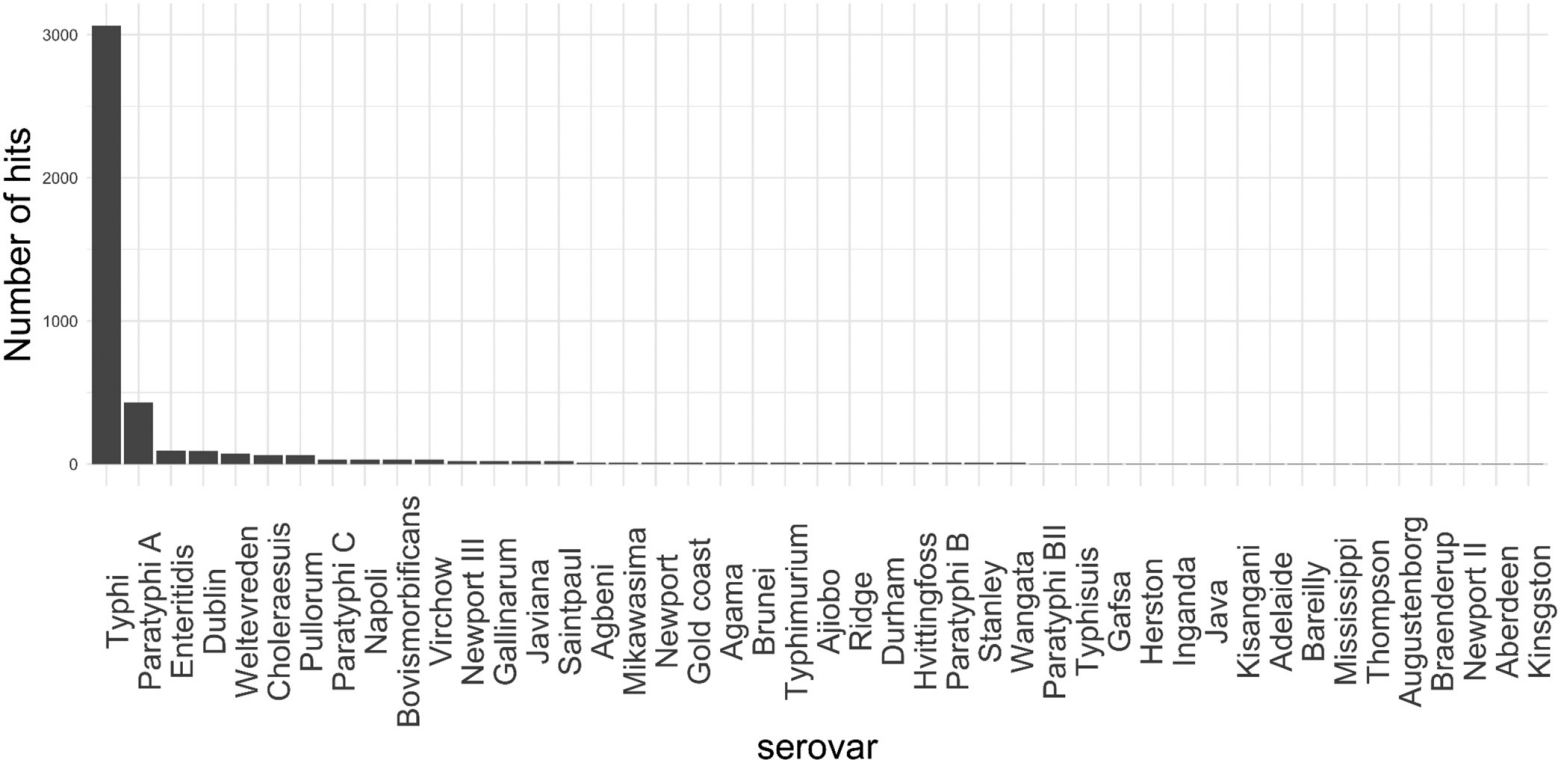
Prevalence of the yreP + regulatory region + yjcS segment in Salmonella serovars based on BLAST hits. The top 10 serovars have been described previously as sources of invasive illness. Serovars containing < 5 isolates were removed from this figure for clarity.

In total, 83% of BLAST hits come from well-known extraintestinal serovars, dominated by S. Typhi strains (Fig. 3). The top 10 serovars in terms of number of hits are mostly invasive serovars: S. Typhi, *S.* Paratyphi A, and *S.* Paratyphi C are notable typhoidal serovars adapted to humans; *S.* Dublin, *S.* Pullorum, and S. Choleraesuis are well-known host adapted serovars of cattle, poultry, and pigs, respectively (4, 11). *S.* Napoli is an emerging serovar in Europe, phylogenetically closely related to *S.* Paratyphi A, carrying an almost identical pattern of typhoid-associated genes, and capable of causing a form of invasive nontyphoidal disease (38, 39). The invasive behavior is not as clear-cut with the rest of the serovars, but there have been several reports of it: *S.* Bovismorbificans is capable of causing bloodstream infections, and has recently been described as an emerging disease in Malawi, converging toward a phenotype resembling a human adapted iNTS variant (40). Although not strictly an extraintestinal serovar, *S.* Virchow has been known to cause invasive illness (41–44). *S.* Weltevreden is an emerging cause of diarrheal and sometimes invasive disease in humans in tropical regions, and may be adapted to life in aquatic hosts (45, 46). While large in total numbers in pubMLST, S. Enteritidis only makes up 2% of the positive hits. Because S. Enteritidis is one of the most commonly isolated iNTS strains, there exists a possible link to invasive behavior (47, 48). However, more work is needed to uncover whether the two proteins are beneficial to an extraintestinal lifestyle.

This brief case study highlights how the information contained in and linked with SalmoNet2 can be used to formulate scientific questions, relating the functionality of genes to the behavior and phylogenetics of Salmonella. SalmoNet2 contains example strains from the most prevalent serovars, and the information can further be extended using the easily accessible sequence data and homology information through OMA and other computational resources.

## DISCUSSION

Here, we present SalmoNet2, a major update and extension of SalmoNet1, offering multilayered interaction networks for 20 Salmonella strains. SalmoNet2 samples a larger diversity of the Salmonella genus, provides a strain-specific metabolic layer, increases the number of high-quality protein-protein interactions, adopts community standard computational formats to help interoperability, and includes online tutorials to help users analyze their data using SalmoNet2.

Multilayered networks enable the user to analyze processes within Salmonella in an integrative manner. Salmonella manipulates host mechanisms to its advantage in a multitude of ways: through its uptake by macrophages carrying it to sites of infection, through the modulation of host metabolism coercing an inflamed state sidestepping competition, or through the ubiquitination of key host proteins (15, 49, 50). Having the ability to find the master regulators directing these processes can aid us in better understanding Salmonella pathogenesis (51, 52). With SalmoNet2 including additional important human-pathogenic Salmonella strains, both typhoidal and nontyphoidal, more targeted analysis is now possible focusing on human disease. Because many of the included extraintestinal serovars have adapted to different host species, eliminating the differences arising from the acclimation of these pathogens to their specific microenvironments could help specialists target the human-disease specific interactions and subgraphs.

With more and more high-quality information available about the Salmonella host-pathogen PPI interface (53), the intracellular regulation of these intercellular interactions can be further investigated with computational tools such as MicrobioLink (54).

SalmoNet2 enables the network analyses such as the use case using rewiring analysis. It highlights how the information contained in and linked with SalmoNet2 can be used to inform scientific questions such as relating the functionality of genes to phenotypes and phylogenetics of Salmonella, based on molecular interaction information.

While the interaction data can be informative on its own, it can be further enhanced and contextualized with the addition of other layers of information, such as results from transcriptomics experiments. Functional transcriptomic resources in Salmonella research such as SalComMac, SalComRegulon or SalComD23580 in the case of S. Typhimurium D23580 can add further detail beyond the interaction structure of networks. Showing how specific subgraphs or network modules behave under various environments and stresses can add dynamics to the network, highlighting active and inactive pathways, regulons (55–57).

Through the development of a more compatible structure between SalmoNet2 and other accessible large-scale evolutionary genomics tools such as OMA, there is enhanced potential to produce interaction networks for specific Salmonella strains on demand, or build analogous data resources for other nonmodel organisms (58). With the implementation of SalmoNet2 with OMA to determine orthologous SalmoNet2 interactions, there are more opportunities to study the evolutionary history of proteins and interactions. As information on orthologous proteins is available beyond the research organism or phylogeny through OMA, larger scale comparisons are possible (59).

The most computationally intensive step of the SalmoNet2 workflow is orthology mapping. Because the all-against-all Smith-Waterman sequence alignments can be parallelized on both single computers or high-performance clusters, the OMA standalone software is considerably faster (18). Adding a new strain or species in the future is also made easier, as OMA Standalone does not require an all-against-all recomputing of the orthologous relationships in these cases, as precomputed results can be submitted, in which case only the new genomes require computation time. Using OMA is not only beneficial for the orthology mapping, it is also helpful for the annotation work. SalmoNet1 is essentially UniProt based, with UniProt IDs serving as the primary identifiers of the database. Currently not all proteins of all strains have a matching UniProt ID, and hence, the OMA IDs are our new primary identifier.

Although there are other resources containing Salmonella interaction data, such as STRING for PPI interactions, RegPrecise for regulatory interactions, or BioCyc for metabolic interactions, no other freely available resource combines the listed connection types besides SalmoNet2, for multiple Salmonella strains (52, 60, 61). In addition to combining different layers of information, we made sure to specify the networks to the individual strains as much as possible, for example, through the removal of strain-specific pseudogenes. This was done under the assumption that these disrupted sequences are no longer functional in these Salmonella strains, but we note that there may be cases where the opposite is true (55, 56).

As part of our update, we adopted the PSI-MI TAB format as well which is quickly becoming a standard of biological network information (12, 57). We also made SalmoNet2 available through the NDEx network repository. This change makes the networks directly accessible from the popular Cytoscape network analysis software, making it easier for end-users to start analyzing SalmoNet2 data (25).

Beyond their raw information content, databases are as good as their usability and availability. For this project to be relevant to the scientific community, the ability to find and use SalmoNet2 data in as many ways as possible is critical (62). To further enhance the accessibility of SalmoNet2 data, we created detailed step-by-step tutorials describing the computational steps required to perform analyses such as the comparisons involving the gastrointestinal and typhoidal strains above. While we extended the number of strains included in SalmoNet2, for the vast majority of sequenced Salmonella genomes, there are no interaction networks available. To address this, we created a tutorial and GitHub repository showing the necessary initial steps to generate custom SalmoNet2 networks for interested researchers, and provided helper scripts to handle the outputs of OMA standalone. For more in-depth steps, interested researchers can consult our previous publication on constructing interaction networks for nonmodel organisms that focused specifically on Salmonella (58).

In summary, we developed a major update to the first biological network resource for Salmonella, SalmoNet2. By increasing the number of available strains compared with SalmoNet1, SalmoNet2 includes information on a member of another subspecies (subspecies *arizonae*), and an entirely different species (Salmonella bongori). The larger evolutionary distance between this additional subspecies and species can further help Salmonella researchers study the evolutionary history of the genus in a new context, and contrast the differences to the more studied human-pathogenic strains (63, 64). In the future, the closer integration of SalmoNet2 with the OMA ecosystem makes genome-to-network pipelines feasible to create. The potential to generate interaction networks on request, or to map Salmonella breakouts, not only through genomics, but comparative network approaches, could be a useful tool in the future for Salmonella studies. In addition, the possibility of generating strain specific networks to characterize the samples of a specific outbreak or epidemic strain could give us further insights into the adaptation of Salmonella to specific environments and stressors by helping specialists fill the gap between the tracked genotypes and the observed phenotypes. By identifying distinct interactions and potentially different substrates and enzyme products between serovars and strains, SalmoNet2 could also provide information for pathogen tracking in the field, as well as laboratory design of methods to isolate a specific serovar in mixed populations, a goal that remains important, but elusive in Salmonella diagnostics.

## MATERIALS AND METHODS

### Updated orthology mapping tool.

Although the main structure of the database remained the same, the underlying workflow changed. As with SalmoNet1, we established the orthologous relationships of proteins across the included Salmonella strains. In SalmoNet1, this was done by InParanoid, an established tool for this process (65). In this update we used the OMA (“Orthologous MAtrix”) standalone software with default parameters to construct these relationships, including the available Salmonella strains from the OMA browser database. OMA is method and database for the inference of orthologs among complete genomes, containing the orthology information and protein sequence data needed for SalmoNet2 in one place, including the proteomes and genomes of the strains on request, and important annotation data (66). We note that at a later database construction step *S.* Pullorum was excluded due to missing data for one of the layers.

The OMA inference algorithm computes an all-against-all alignment to find homologs between all the included proteins, resulting in pairwise orthologs. These pairwise orthologous relationships are mapped on a graph, from which the “OMA Groups” are derived. OMA groups are cliques of the orthology graph; in other words, members of an OMA group are all connected to each other through pairwise orthologous relationships. Further details of the algorithm can be found in the OMA algorithm publications (67, 68).

It is important to note, that the outputs of the tools can be slightly different: according to a study comparing orthology inference methods the OMA standalone output, “OMA groups” led to a generally more precise, but also strict mapping, leading to increased specificity but decreased sensitivity (69). We did, however, get very similar, and in some cases better recall, than we did in SalmoNet1 (between 69 and 75% overlap with the 4,140 proteins from E. coli*;* Table S1) using InParanoid.

### Updated and novel data sources.

**(i) Protein-protein interaction networks.** The construction of the PPI network follows the same essential steps it did in the first version of the database, carrying over the same interaction sources, such as BioGrid, Interactome 3D, and data from low- and high-throughput experiments (70–72). We amended our earlier methodology to increase the coverage of the included PPIs without losing quality from the IntAct database. We employed the IntAct PSI-MIscore (>0.50) when importing interaction information from the IntAct database, instead of relying on one experimental method, as in the first version (psi-mi:“MI:0096”[pull down]). The IntAct PSI-MIscore is based on the manual annotation of every instance of a binary interaction (A to B) within the IntAct database, where interactions are scored based on interaction detection method, interaction type, and the number of publications the interaction has appeared in, normalized between 0 and 1. Fig. S1 shows the distribution of the IntAct PSI-MIscores, and the corresponding IntAct PSI-MIscores are shown on the website when applicable.

**(ii) Metabolic networks.** SalmoNet2 uses new, strain specific genome-scale metabolic models for Salmonella (22, 23). The novel input models used the same STM 1.0 model as the starting point as used in SalmoNet1 (21) but were updated with additional genes and reactions, and were made strain specific, leading to the metabolic models of 410 Salmonella strains belonging to 64 serovars. Otherwise, the workflow remained identical, identifying enzyme-enzyme interactions, where two proteins are connected if a metabolite produced by one is a substrate for another (73). Similarly as in SalmoNet1, we have excluded links connected by metabolites partaking in more than 10 reactions (73). To help interoperability with external data sets and improve the annotation status of metabolic enzymes, we have mapped strain-specific KEGG identifiers to them where this was available. In cases where no strain-specific KEGG annotation data were available, we used the orthologous KEGG identifiers from S. Typhimurium LT2, while also indicating that the source of the KEGG identifier is based on orthology (“ort:” prefix).

**(iii) Regulatory networks.** The establishment of the transcriptional regulatory networks was done in an identical way to SalmoNet1. Fig. S2 shows the workflow for the construction of the regulatory layer. The core of the network, the manually curated interactions, high-throughput data (ChIP-Seq), and low-throughput, experimentally verified interactions and data sources remained the same and carried over from SalmoNet1. The information content of PSSMs used to carry out the genome-wide scans was enhanced with novel binding sites published since the first version of the database, from new data uploaded to the CollecTF repository (24). As RSAT’s consensus tool is no longer available on the web server and info-gibbs took its place, the latter method was used to construct the PSSMs. Similarly, as previously, RSAT retrieve-sequence was used to gather the putative promoter regions for the genomes included in SalmoNet2, and matrix-scan was used to establish putative transcription factor-target gene (promoter region) pairs (74).

10.1128/msystems.01493-21.3 FIG S2 Updated workflow used for the construction of the transcriptional regulatory layer of interactions. Download FIG S2, PDF file, 0.1 MB. Copyright © 2022 Olbei et al. 2022 Olbei et al. https://creativecommons.org/licenses/by/4.0/ This content is distributed under the terms of the Creative Commons Attribution 4.0 International license.

**(iv) Phylogenetic trees and network dendrograms.** For the phylogenetic tree core genome, SNPs were determined with snippy (version: 4.3.6), with the snippy-multi and snippy-core functions ran on the Earlham Institute High Performance Cluster (https://github.com/tseemann/snippy). MegaX was used to build a newick tree file from the resulting core genome SNP alignment. All trees were visualized using the ggtree R language package (75, 76).

The network dendrograms were generated using a Metropolis coupling Markov Chain Monte Carlo (MC3) from the MrBayes (version: 3.2.4) software with 10 million generations; 25% of the samples were discarded during the MrBayes run. To accommodate the binary data, the data type was set to restriction, and no substitution model was used (77). This is identical to the approach that was used to generate network based dendrograms for SalmoNet1 (11).

The PERMANOVA analysis of the dendrograms was carried out with the R package vegan using the adonis2 function with the method “bray” and 10,000 permutations. The scripts used for the PERMANOVA analysis can be found in the project GitHub repository (https://github.com/korcsmarosgroup/SalmoNet2).

### Removal of pseudogenes.

To remove all hypothetically disrupted coding DNA sequences (HDCs), the curation made by Nuccio and Bäumler was used to remove such entries (15), and references 78 and 79 were used to remove them from S. Typhimurium D23580. This was done under the assumption that these disrupted sequences are no longer functional in these strains.

### Network rewiring.

To calculate network rewiring we used the DyNet app in Cytoscape to calculate the rewiring value of the nodes in each group separately (29). DyNet identifies the most dynamically changing, or most rewired neighborhoods between the compared networks.

Four host adapted typhoidal strains (*S.* Paratyphi A [AKU 1261], *S.* Paratyphi A [ATCC 9150], *S.* Paratyphi C [RKS4594], S. Typhi [Ty2]) and four gastrointestinal strains (*S.* Agona [SL483], *S.* Newport [SL254], *S.* Heidelberg, S. Typhimurium [LT2]) were compared for interaction differences. The level of rewiring was calculated across all strains, and the degree-corrected rewiring values were ordered in a descending list, where the top 50 hits were further analyzed.

To calculate the enrichment of Gene Ontology terms in the identified subgraphs the up-to-date Gene Ontology annotation of the target, genes was downloaded using the topGO library in R, and then the R library clusterProfiler was used to calculate Gene Ontology enrichment with the enricher function, from Biological Process terms (80, 81). *P*-value adjustment for multiple testing was carried out with the Benjamini-Hochberg approach, using the p.adjust function in R.

The statistically significant enrichment results were compared side-by-side between the groups, and the differences in enrichment were further studied by comparing the sets of genes responsible for (underlying) the enriched terms, i.e., if one group was enriched in a specific term, the presence/absence of the orthologous genes responsible for the enrichment was analyzed in the members of the other group.

To study the relationship of YreP and YjcS to the extraintestinal pathovar, network rewiring was calculated in an identical manner as above, but all extraintestinal and gastrointestinal strains from SalmoNet2 were used for the comparisons. BLAST searches for the *yreP* and *yjcS* genes was done through the pubMLST website, with default parameters (37). The entire genomic sequence of the genes and their shared regulatory region was queried, as taken from *S.* Gallinarum strain 287/91 (see Text S1). The hits were filtered for over or greater than 95% sequence identity, and the top 10% of bitscores to make sure the compared sequences contain both the genes and the shared regulatory region.

10.1128/msystems.01493-21.4 TEXT S1 Nucleotide FASTA of region used for BLAST search. Download Text S1, TXT file, 0.04 MB. Copyright © 2022 Olbei et al. 2022 Olbei et al. https://creativecommons.org/licenses/by/4.0/ This content is distributed under the terms of the Creative Commons Attribution 4.0 International license.

### Data availability.

The data generated for this study is available at the database website, http://salmonet.org.
