# Supplementary material for: Multilayered Networks of SalmoNet2 Enable Strain Comparisons of the Salmonella Genus on a Molecular Level
Source: mSystems. 2022 Aug 1;7(4):e01493-21. doi: 10.1128/msystems.01493-21 (PMC9426430; doi:10.1128/msystems.01493-21)
Supplement: TABLE S1 [file msystems.01493-21-s0002.pdf]

Strain	Five letter code	Orthologous protein overlap with *E. coli*	Percentage match
Salmonella enterica subsp. *Enterica* serovar *Agona* str. *SL483*	SALA4	3016	72.8%
Salmonella enterica subsp. *Arizonae* serovar *62:z4,z23:-*	SALAR	2859	69.1%
Salmonella bongori NCTC 12419	SALBC	2961	71.5%
Salmonella enterica subsp. *Enterica* serovar *Choleraesuis* str. *SC-B67*	SALCH	2987	72.1%
Salmonella enterica subsp. *Enterica* serovar *Dublin str. CT 02021853*	SALDC	2983	72.1%
Salmonella enterica subsp. *Enterica* serovar *Enteritidis* str. *P125109*	SALEP	3092	74.7%
Salmonella enterica subsp. *Enterica* serovar *Gallinarum* str. *287/91*	SALG2	3075	74.3%
Salmonella enterica subsp. *Enterica* serovar *Heidelberg* str. *SL476*	SALHS	3044	73.5%

Salmonella enterica subsp. *Enterica* serovar *Newport* str. *SL254*	SALNS	3033	73.3%
Salmonella enterica subsp. *Enterica* serovar *Paratyphi A* str. *AKU 12601*	SALPK	3006	72.6%
Salmonella enterica subsp. *Enterica* serovar *Paratyphi A* str. *ATCC 9150*	SALPA	2960	71.5%
Salmonella enterica subsp. *Enterica* serovar *Paratyphi B* str. *SPB7*	SALPB	3077	74.3%
Salmonella enterica subsp. *Enterica* serovar *Paratyphi C str. RKS4594*	SALPC	2996	72.3%
Salmonella enterica subsp. *Enterica* serovar *Schwarzengrund* str. *CVM19633*	SALSV	2993	72.3%
Salmonella enterica subsp. *Enterica* serovar *Typhimurium* str. *14028S*	SALT1	3109	75.1%
Salmonella enterica subsp. *Enterica* serovar *Typhimurium* str. *LT2*	SALTY	3103	74.9%

Salmonella enterica subsp. *Enterica* serovar *Typhimurium* str. *ST4/74*	SALT4	3110	75.1%
Salmonella enterica subsp. *Enterica* serovar *Typhimurium* str. *SL1344*	SALTS	3107	75%
Salmonella enterica subsp. *Enterica* serovar *Typhimurium* str. *D23580*	SALTD	3095	74.8%
Salmonella enterica subsp. *Enterica* serovar *Typhi* str. *CT18*	SALTI	3013	72.8%
