# Supplementary figures and images for: Multilayered Networks of SalmoNet2 Enable Strain Comparisons of the Salmonella Genus on a Molecular Level
Source: mSystems. 2022 Aug 1;7(4):e01493-21. doi: 10.1128/msystems.01493-21 (PMC9426430; doi:10.1128/msystems.01493-21)

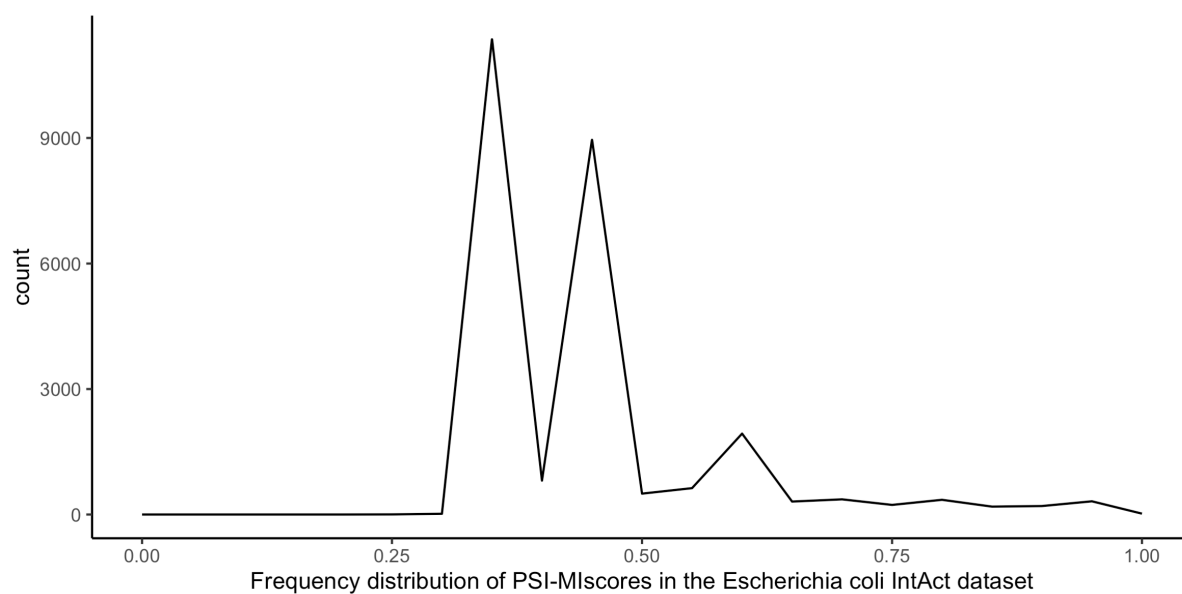

Supplement: FIG S1 [file msystems.01493-21-s0001.pdf]

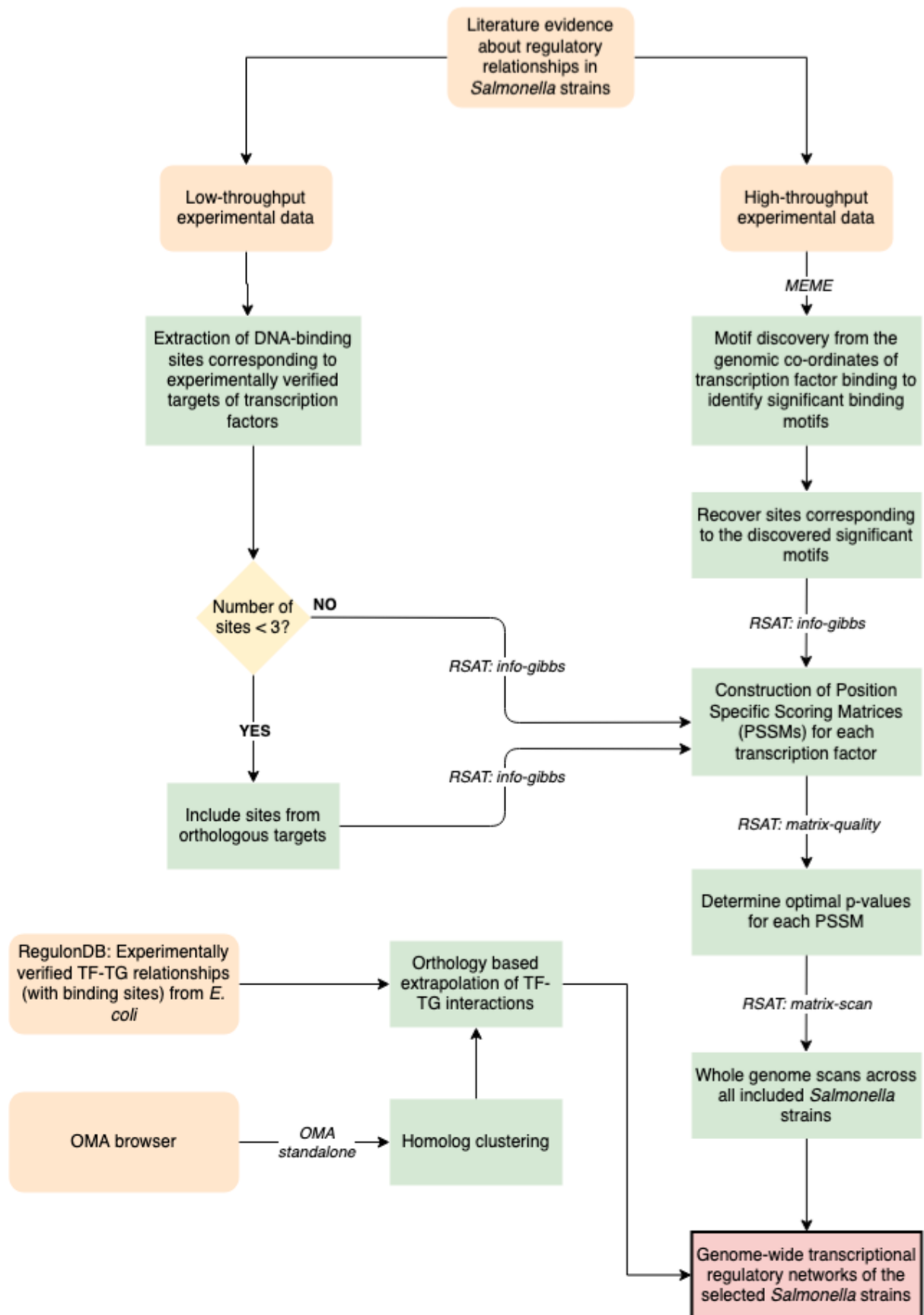

Supplement: FIG S2 [file msystems.01493-21-s0003.pdf]
